# DUETT quantitatively identifies known and novel events in nascent RNA structural dynamics from chemical probing data

**DOI:** 10.1093/bioinformatics/btz449

**Published:** 2019-08-07

**Authors:** Albert Y Xue, Angela M Yu, Julius B Lucks, Neda Bagheri

**Affiliations:** 1 Department of Chemical & Biological Engineering, Northwestern University, Evanston, IL, USA; 2 Center for Synthetic Biology, Northwestern University, Evanston IL, USA; 3 Northwestern Institute on Complex Systems, Northwestern University, Evanston, IL, USA; 4 Tri-Institutional Training Program in Computational Biology and Medicine, Weill Cornell Medicine, New York, NY, USA; 5 Chemistry of Life Processes Institute, Northwestern University, Evanston, IL, USA

## Abstract

**Motivation:**

RNA molecules can undergo complex structural dynamics, especially during transcription, which influence their biological functions. Recently developed high-throughput chemical probing experiments that study RNA cotranscriptional folding generate nucleotide-resolution ‘reactivities’ for each length of a growing nascent RNA that reflect structural dynamics. However, the manual annotation and qualitative interpretation of reactivity across these large datasets can be nuanced, laborious, and difficult for new practitioners. We developed a quantitative and systematic approach to automatically detect RNA folding events from these datasets to reduce human bias/error, standardize event discovery and generate hypotheses about RNA folding trajectories for further analysis and experimental validation.

**Results:**

Detection of Unknown Events with Tunable Thresholds (DUETT) identifies RNA structural transitions in cotranscriptional RNA chemical probing datasets. DUETT employs a feedback control-inspired method and a linear regression approach and relies on interpretable and independently tunable parameter thresholds to match qualitative user expectations with quantitatively identified folding events. We validate the approach by identifying known RNA structural transitions within the cotranscriptional folding pathways of the *Escherichia coli* signal recognition particle RNA and the *Bacillus cereus crcB* fluoride riboswitch. We identify previously overlooked features of these datasets such as heightened reactivity patterns in the signal recognition particle RNA about 12 nt lengths before base-pair rearrangement. We then apply a sensitivity analysis to identify tradeoffs when choosing parameter thresholds. Finally, we show that DUETT is tunable across a wide range of contexts, enabling flexible application to study broad classes of RNA folding mechanisms.

**Availability and implementation:**

https://github.com/BagheriLab/DUETT.

**Supplementary information:**

Supplementary data are available at *Bioinformatics* online.

## 1 Introduction

**Fig. 1.**
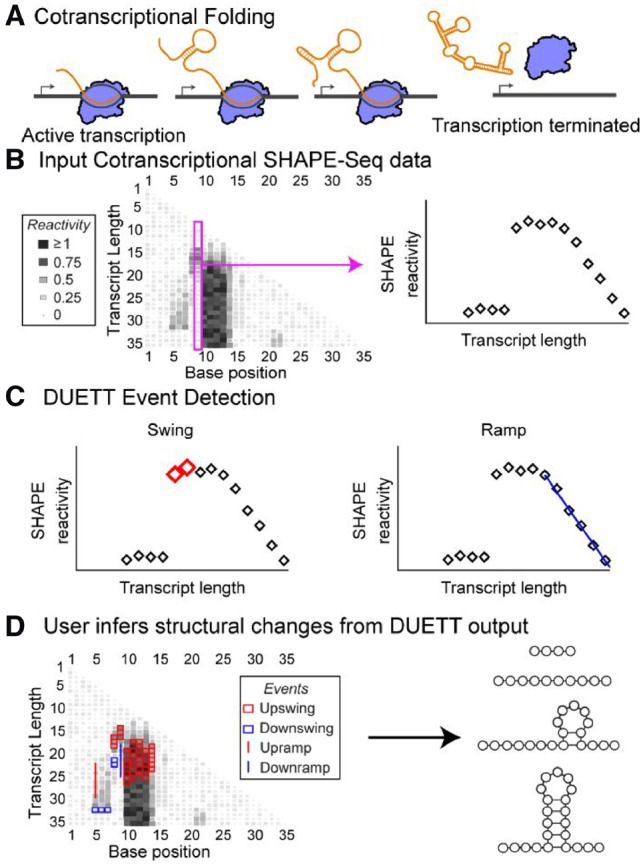
DUETT provides an automated and systematic method to detect cotranscriptional RNA folding events from SHAPE-Seq data. (**A**) RNA can dynamically alter structure during transcription that affects downstream biological functions. (**B**) Cotranscriptional SHAPE-Seq probes RNA structural properties during transcription by measuring reactivity patterns for each intermediate length of an RNA. High and low reactivity correspond to unstructured and constrained regions of RNA, respectively. (**C**) DUETT is a flexible method to identify large and gradual reactivity changes that are indicative of RNA structural transitions that can happen between intermediate RNA lengths. (**D**) Here DUETT is applied to a mock dataset to identify changes in reactivities in several consecutive nucleotides corresponding to the formation of an RNA loop, which is consistent with the typical ‘low-high-low’ pattern observed for RNA hairpin structures

RNA molecules play diverse functional roles ranging from catalysis of splicing and peptide bond formation, regulation of mRNA processing and gene expression, and molecular scaffolding and localization (Cech and Steitz, 2014; Sharp, 2009). These functions are in turn mediated by RNA structures that form in complex cellular environments. RNA structures are diverse and can prohibit or promote interactions with other RNAs, proteins and metabolites to enable a broad range of RNA function. For example, bacterial RNA structures inhibit transcription elongation (Ray-Soni *et al.*, 2016), translation initiation (Afonin *et al.*, 2016) and RNA degradation (Hui *et al.*, 2015). In eukaryotes, there is growing evidence that RNA structure impacts gene expression processes (Ding *et al.*, 2014; Rouskin *et al.*, 2014; Spitale *et al.*, 2015; Talkish *et al.*, 2014). However, we know little about how newly synthesized, or nascent RNAs fold during transcription (Pan and Sosnick, 2006; Woodson, 2010). Due to the relative timescales of RNA folding and transcription, RNA molecules begin to fold as they emerge from RNA polymerase (RNAP) (Dethoff *et al.*, 2012) (Fig. 1A). RNAs can transition between states in this cotranscriptional folding pathway that dictate RNA folding and function. For example, riboswitches dynamically alter their structure during transcription in response to ligand binding, leading to ligand-dependent structural, and regulatory switching (Smith *et al.*, 2009). In addition, there is emerging evidence that cotranscriptionally-formed RNA structures can influence various processes in eukaryotes, such as splicing (Shukla and Oberdoerffer, 2012) and 3' end processing of histone mRNAs (Saldi *et al.*, 2016). There has been great interest in developing both computational and experimental approaches to uncover RNA cotranscriptional folding pathways and their implications for cellular RNA function.

Recently developed experimental techniques can characterize cotranscriptional RNA folding at nucleotide resolution (Strobel *et al.*, 2017; Watters *et al.*, 2016b) by utilizing high-throughput chemical probing of RNA structure (Strobel *et al.*, 2018). SHAPE reagents (selective 2'-hydroxyl acylation analyzed with primer extension) are chemical probes that form adducts at the 2'-OH of each nucleotide (Merino *et al.*, 2005). When coupled with high-throughput sequencing, SHAPE experiments reveal detailed reactivity patterns that uncover RNA structural properties—highly-reactive positions indicate lack of structure and lowly reactive positions indicate constraint due to structure or interaction with other binding partners (Bindewald *et al.*, 2011; Steen *et al.*, 2010; Watters *et al.*, 2016a). An experimental variant called cotranscriptional SHAPE-Seq probes the structure of each intermediate length RNA during transcription (Fig. 1) (Strobel *et al.*, 2017; Watters *et al.*, 2016b). This experiment results in a matrix of reactivities, where rows correspond to reactivity at each length of a growing nascent RNA chain, and columns represent reactivity changes at specific nucleotides across different RNA lengths (Fig. 1B). Both dimensions of the reactivity matrix reflect possible changes in RNA structural state that can occur during transcription. For example, a decrease (or increase) in reactivity down a column highlights a possible folding (or unfolding) event during transcription.

However, analysis of the cotranscriptional reactivity matrices has been mostly qualitative, relying on manual identification of reactivity trends to identify key regions that have biological significance. As the number and complexity of these datasets grow, quantitative and automated techniques are needed to robustly identify patterns. This automated quantitative approach is challenging, as cotranscriptional SHAPE-Seq datasets with complete annotations and validated structures are not bountiful. This scarcity of ‘ground truth’ examples presents difficulties when defining statistical models (Hogenboom *et al.*, 2011) and prohibits application of machine learning models (Margineantu *et al.*, 2010), which have been implemented in other applications such as emulating visual detection of meaningful reactivities as labeled by experimental experts (Woods and Laederach, 2017). Similarly, interpretation of labeled datasets are prone to human error and subjectivity; depending upon expertise or prior expectations of RNA folding events, different viewers may disagree on interpretations of subtle reactivity changes. In addition, SHAPE-Seq reactivities differ with RNA structure and experimental context, requiring a tunable computational approach. For example, a significant reactivity change in a low-reactivity dataset can appear as noise in a high-reactivity dataset. These limitations suggest that we require a systematic method to identify signatures of RNA structural dynamics from cotranscriptional reactivity datasets that use interpretable, user-guided rules.

To overcome this challenge, we sought to develop a quantitative and automated approach to identify trends in cotranscriptional SHAPE-Seq datasets. We designed a systematic detection method that remains user-tunable with an interpretable set of parameters to easily match qualitative user expectations with quantitatively identified folding events. Other computational approaches have successfully emulated subjective human-driven analyses, such as extracting RNA design rules from a crowd-sourced game (Lee *et al.*, 2015). Due to the complexity of RNA structures and the flexibility of SHAPE-Seq applications/implementations, we opted to detect generic events. This philosophy is common in domains with poorly defined events, such as identifying unknown genomic deletions and insertions (Jiang *et al.*, 2015; Ye *et al.*, 2009).

We present a framework for detecting events in cotranscriptional SHAPE-Seq datasets termed Detection of Unknown Events with Tunable Thresholds (DUETT). DUETT detects swing events using a strategy inspired by proportional-integral feedback control (Florian and Parker, 2007) and detects ramp events using linear regression. Swing events represent rapid reactivity changes that occur over a small number of transcript lengths. In contrast, ramp events represent slower changes that span many transcript lengths. DUETT provides automated threshold parameter optimization, but DUETT also allows user-defined parameter tuning to match a wide range of experimental contexts. We first define these methods and identify parameters that robustly identify known folding events within the cotranscriptional folding pathway of the *Escherichia* *coli* signal recognition particle (SRP) RNA. We extend the methodology to analyze the folding pathways of the *Bacillus* *cereus crcB* fluoride riboswitch and corroborate previous manually identified transitions. In both datasets, our analysis reveals unexpected behavior, such as subtle reactivity increases that consistently occur roughly 12 nt lengths before a reactivity decrease, suggesting a highly-reactive transient state. Finally, we conduct parameter sensitivity analysis to explore the relationship between DUETT’s parameter values and detected events. The flexibility and interpretability of our approach enables the broad application of DUETT to many high-throughput experimental systems that require event detection.

## 2 Materials and methods

### 2.1 Event detection

Structural events are characterized by significant reactivity changes at specific nucleotide positions, across sequential transcript lengths. We consider two common, yet distinct, phenomena in cotranscriptional SHAPE-Seq datasets: swing and ramp events (Fig. 1). These two qualitatively different classes of events motivate separate detection methods for each event type: PIR-control and linear regression, respectively (Supplementary Fig. S1). Full DUETT methods and motivations are located in Supplementary Materials. Assumptions are explicitly listed alongside their design consequences in Supplementary Table S1. Briefly, DUETT uses proportional-integral feedback control to detect swing events and uses linear regression to detect ramp events.

### 2.2 Simulation of cotranscriptional SHAPE-Seq data

We simulated an expected cotranscriptional folding pathway of an *in silico* RNA by first using NUPACK (Zadeh *et al.*, 2011) to design RNA sequences that fold into a desired intermediate and final structure. Expected structures of each intermediate transcript length of this sequence were predicted from RNAstructure-Fold (Reuter and Mathews, 2010). SHAPE reactivities for each structure were simulated using a previously published ternary model (Sükösd *et al.*, 2013) with 14 additional paired nucleotides 3' appended to the end of the structure in order to emulate the protection from SHAPE modification due to the RNAP footprint. Simulation of SHAPE reactivities were performed 1000 times and averaged. Finally, simulated SHAPE reactivities for each length were renormalized to a mean of 1, mimicking the *ρ* reactivity measure.

### 2.3 Application to cotranscriptional SHAPE-Seq datasets

We applied DUETT to simulated data and two RNA sequences characterized by previous cotranscriptional SHAPE-Seq experiments (Watters *et al.*, 2016b): the *E.coli* SRP RNA and the *B.cereus crcB* fluoride riboswitch. These published datasets were obtained from the Small Read Archive (http://www.ncbi.nlm.nih.gov/sra) with the BioProject accession code PRJNA342175. The data were processed with Spats v1.01 (https://github.com/LucksLab/spats/releases/) and the reactivity calculation scripts are located at https://github.com/LucksLab/Cotrans_SHAPE-Seq_Tools/releases/.

## 3 Results

We validated DUETT by identifying known cotranscriptional structural events that occur in the folding pathways of three RNA molecules: a simulated dataset on a synthetic RNA sequence, the *E.coli* SRP RNA and the *B.cereus* fluoride riboswitch (Batey *et al.*, 2000; Watters *et al.*, 2016b; Wong *et al.*, 2007). We used the automated approach to select *PIR* threshold parameters and manually selected the same linear ramp thresholds across all datasets. During the automated search, the increasing *PIR* thresholds cause the number of detected events to rapidly decrease until reaching an elbow (Supplementary Fig. S2). The point closest to the origin lies near the elbow and corresponds to the optimized threshold values, similar to how numbers of clusters are chosen in clustering algorithms (Gao *et al.*, 2018). We applied DUETT on each of three experimental replicates for each system and retained events conserved across all replicates to decrease the chance of spurious events. This approach creates similar results as applying DUETT to the average of all replicates (Supplementary Fig. S3) but avoids scenarios where a single replicate has anomalous values. DUETT identified both known and novel structural events, and we propose novel hypotheses for further study. We discover patterns and events that are difficult for a human to identify. We conclude with parametric sensitivity analysis to explore the relationship between user-defined threshold parameters and observed events, and a discussion of limitations of experimental datasets.

### 3.1 Validation on simulated cotranscriptional SHAPE-Seq data

Benchmarking on simulated cotranscriptional SHAPE-Seq data was performed to establish how accurately DUETT detects expected structural changes (Supplementary Fig. S4). Qualitatively, the simulated data resulted in sharper reactivity changes between structured and unstructured nucleotides than in previously published cotranscriptional SHAPE-Seq data (Supplementary Figs S5, S7 and S9). However, this simulated data provides expected structures throughout the folding pathway and was thus ideal for testing DUETT’s ability to detect expected structural changes.

When applied to the simulated dataset, DUETT picks up events corresponding to major structural changes (Supplementary Fig. S4). We analyzed three categories of expected upswing/downswing events in aggregate: upswings due to nucleotides exiting the RNAP footprint and becoming unpaired, downswings when nucleotides transition from unpaired to paired, and upswings when nucleotides transition from paired to unpaired. By visual inspection, there are 42 nts with a RNAP footprint to unpaired transition that should result in upswings. DUETT detected 27 of these transitions with the missing events resulting from too few preceding transcript lengths such as with nucleotides 1–12. Other missed events include short- low-high-low transitions such as in nucleotide 51 which pairs shortly after emerging from the RNAP footprint. Similarly, there are ∼45 unpaired to paired event transitions that should result in downswings. DUETT detected 28 downswings but missed events due to proximity to detected upswings, e.g. nucleotide 50 at length 67, as well as transitions that are too short to qualify as an event, e.g. nucleotide 3 at length 82. Finally, there are 17 paired to unpaired transitions, and DUETT only missed two events that were helix-end nucleotides with a smaller reactivity change.

Overall, DUETT identified the major structural changes despite sporadically missing events at individual nucleotides as can easily be observed from looking at these events annotated on the simulated data matrix (Supplementary Fig. S4). We reason that most structural changes involve multiple nucleotides and DUETT has a high likelihood to detect some but not all relevant nucleotides. For example, DUETT detected the pairing of nucleotides 1–5 at transcript length 46 but missed the adjacent helix end of nucleotide 6. This result demonstrates that transitions to and from helix-ends are not easily captured due to the decreased magnitude of reactivity change compared to stacked bases. Similarly, DUETT performs well when events are not close in transcript length to previously found events or the RNAP footprint, especially since the RNAP footprint was simulated as paired nucleotides so reactivities and would be indistinguishable from truly paired nucleotides.

### 3.2 Validation on *E.coli* SRP RNA cotranscriptional SHAPE-Seq datasets and identification of previously unidentified reactivity patterns

**Fig. 2.**
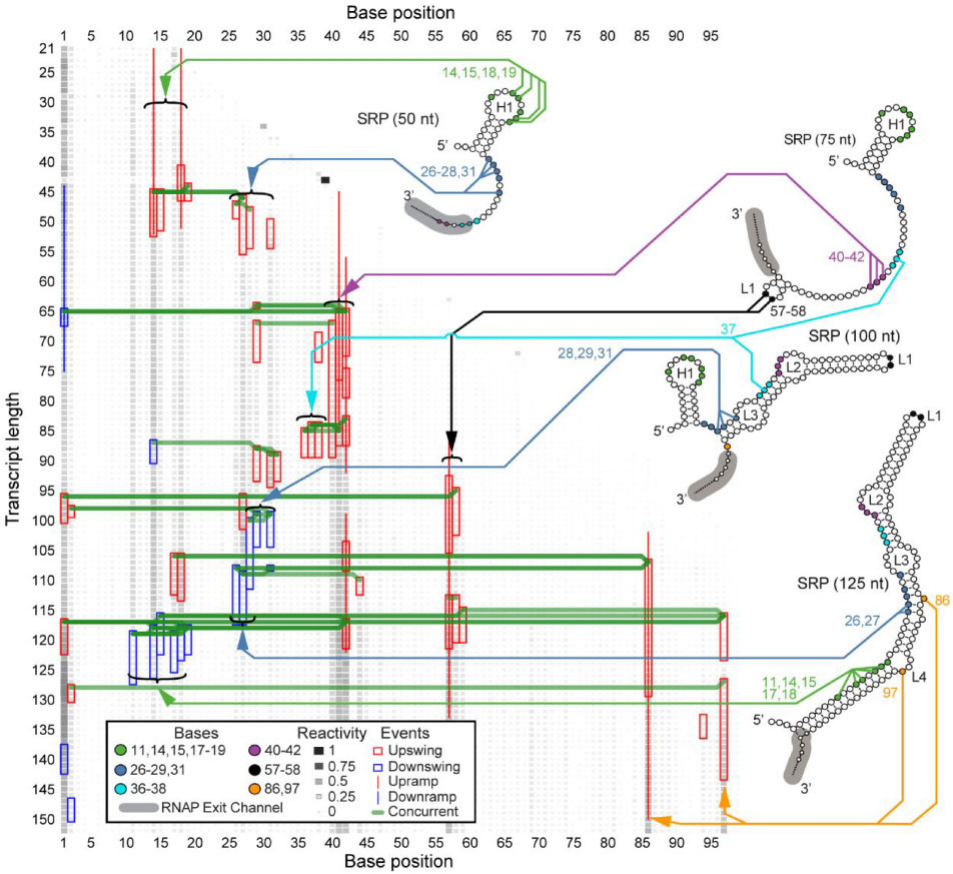
DUETT identifies known RNA folding events in the *E.coli* SRP RNA cotranscriptional SHAPE-Seq reactivity matrices. Four previously proposed intermediate structural conformations of SRP RNA are shown with arrows linking specific color-coded bases to identified reactivity changes. RNA structures are redrawn from Figure 2 of Watters *et al.* (2016b) with intermediate hairpin H1 and loops L1–L4 labeled, and the RNAP exit channel footprint annotated in gray. DUETT identifies multiple instances of hairpin formation/rearrangement and previously unidentified events. DUETT displays detected swing and ramp events as a colored box and line, respectively, with red and blue denoting reactivity increase and decrease events, respectively. A green line connects concurrent events between different nucleotides. SHAPE reactivity is normalized to lie in between the range 0–1 and shown in grayscale and box area

Previous studies have shown that during transcription, the *E.coli* SRP RNA forms a transient 5' hairpin (H1) that rearranges into a long helical structure with a hairpin loop and multiple inner loops (Batey *et al.*, 2000; Watters *et al.*, 2016b; Wong *et al.*, 2007), which we label L1–L4 (Fig. 2). Several of these transitions have been validated by prior bulk studies (Watters *et al.*, 2016b; Wong *et al.*, 2007), and by single molecule optical trapping experiments (Fukuda *et al.*, 2018). A previous analysis of cotranscriptional SHAPE-Seq datasets (Watters *et al.*, 2016b) focused on the manual annotation of patterns within the reactivity matrix to derive a model of the cotranscriptional folding pathway. We thus sought to apply DUETT to these datasets to automatically identify reactivity changes that are reflective of these structural transitions.

#### 3.2.1 DUETT identifies expected H1 formation and rearrangement

The major cotranscriptional rearrangement event for the *E.coli* SRP RNA occurs when H1 refolds into the final extended structure (Fukuda *et al.*, 2018; Yu *et al.*, 2018). Corresponding to the formation of H1, DUETT identified upswings in bases 14–15 and 18–19 around nascent RNA length 45 nt, and upramps that conclude around length 50 nt (Fig. 2). These positions remain unpaired in the intermediate H1 hairpin, validating the correspondence between upswings/upramps and the formation of unpaired, reactive regions. This hairpin rearranges into the long helical structure, which DUETT identifies as downswing events between lengths 116 and 127 nt in bases 11, 14–15 and 17–19. These identifications are consistent with recent computational modeling (Yu *et al.*, 2018) and single molecule optical tweezer experiments (Fukuda *et al.*, 2018) that propose the rearrangement of H1 to occur in the window that DUETT detects.

#### 3.2.2 Multiple expected reactivity changes further validate DUETT

Another key feature of the *E.coli* SRP RNA cotranscriptional folding pathway is the formation of native base pairs and loops after the formation of H1, but before the rearrangement of H1 into the final native structure (Fukuda *et al.*, 2018; Watters *et al.*, 2016b; Yu *et al.*, 2018). DUETT identifies expected reactivity signatures of bases 26–29 and 31 through length 100 nt. Upon initial transcription, bases 26–28 and 31 have upswings corresponding to their unpaired state, and bases 28–29 and 31 have downswings at 100 nt that agree with the previously proposed 100 nt structure in which these nucleotides are paired (Watters *et al.*, 2016b). Identification of other unpaired positions provides additional validation of DUETT event detection. A cluster of upramps/upswings in bases 40–42 between lengths 55 and 90 nts corresponds to the unpaired, interior loop region in L2. Finally, bases 57–58, 86 and 97 have upramps/upswings immediately after transcription that corroborates their unpaired status as the apex nucleotides in the hairpin loop of the final rearranged structure, or within internal loops and bulges, respectively.

#### 3.2.3 Unexpected events highlight overlooked structural dynamics

While DUETT identified previously validated and observed reactivity changes within the SRP RNA cotranscriptional folding pathway, it also identified novel and unexpected events such as a downswing in base 14 and upswings in bases 36–38 at lengths 87 and 84 nts, respectively. These events are discordant with the previously proposed folding model of the SRP RNA which proposes that base 14 remains unpaired in H1 and bases 36–38 pair with bases 74–76 between lengths 75–100 nts. However, these detected events do not appear spurious. The downswing in base 14 is concurrent with other undetected downswings in neighboring bases 11 and 15 (Supplementary File 1 and Fig. S6), and the upswings in bases 36–38 are concurrent and qualitatively similar with one another. These unexpected events occur in the transition that forms the L1 and L2 loops and suggests a transient state that causes decreased reactivity in base 14 and increased reactivity in bases 36–38.

In addition, base 40 was reported to be paired by length 100 nt (Watters *et al.*, 2016b) that corresponds to an undetected downswing at 94 nt (Supplementary File 1). Though base 40 has lower reactivity than bases 41–42 (Supplementary File 1), its reactivity is higher than expected for a base pair. Our DUETT results suggest that U40 is more labile than previously reported (Watters *et al.*, 2016b). We attribute this accessibility to U40’s position at a helix end as well as part of a GU pair with G72 in the native structure and these features are known to be generally less stable (Jaeger *et al.*, 1989; Papanicolaou *et al.*, 1984).

Another unexpected set of events include the downswings of bases 26–27 at length 108nt, which is earlier than the stable rearrangement of H1 into the final helical structure. These downswings in bases 26–27 are concurrent with two upswing events at bases 44 and 86 and an upramp in base 86. Base 44’s upswing is more pronounced in one replicate but is present in all three replicates (Supplementary File 1), and base 86 is a bulged nucleotide in the native structure that forms opposite of base pairs involving positions 27–28. This concurrency suggests that the bulge formation in base 86 occurs simultaneously with bases 26–27 pairing and agrees with the previously proposed model (Watters *et al.*, 2016b).

We also observed unforeseen upswings about 12 nt lengths before downswings, suggesting a highly-reactive transient state. Bases 17–18, 27, 29 and 31 exhibit upswings roughly 12 nt lengths before their downswing events (Fig. 2). The order in which these downswings occur is consistent with the order of transcription, suggesting an order of folding events based on initial exposure and transcription. Coincidentally, when bases 26–27 or bases 29–31 undergo pairing interactions, the preceding upswing pattern occurs in the 3' base(s) of that group. This is similar to the preceding upswing followed by downswing behavior in bases 17–18 and suggests that increased flexibility in the 3' side transiently occurs before becoming stabilized. We note the difficulty in manually detecting these patterns of events, justifying our automated and systematic approach. These observations lead us to believe that the detected events are not spurious, lack an explanation by previously published studies, and highlight the discoveries enabled by our systematic method.

### 3.3 DUETT identifies known and novel structural transitions in the cotranscriptional folding pathway of a fluoride riboswitch

We next sought to determine if DUETT could identify events in the *B.cereus* fluoride riboswitch cotranscriptional SHAPE-Seq data (Watters *et al.*, 2016b). The *B.cereus* fluoride riboswitch is an RNA sequence that lies in the 5' untranslated region of the *crcB* gene and cotranscriptionally folds into mutually exclusive structures that regulate downstream transcription depending upon whether the fluoride anion is bound (Baker *et al.*, 2012). Previous cotranscriptional SHAPE-Seq experiments were done with either 10 or 0 mM NaF conditions. Manual analysis revealed distinct reactivity patterns that are reflective of ligand binding, and the bifurcation of the folding pathway in a fluoride-dependent manner. We thus applied DUETT to both conditions to identify both known and potentially novel RNA folding events.

#### 3.3.1 Similarity of events before length 69 nt between conditions

**Fig. 3.**
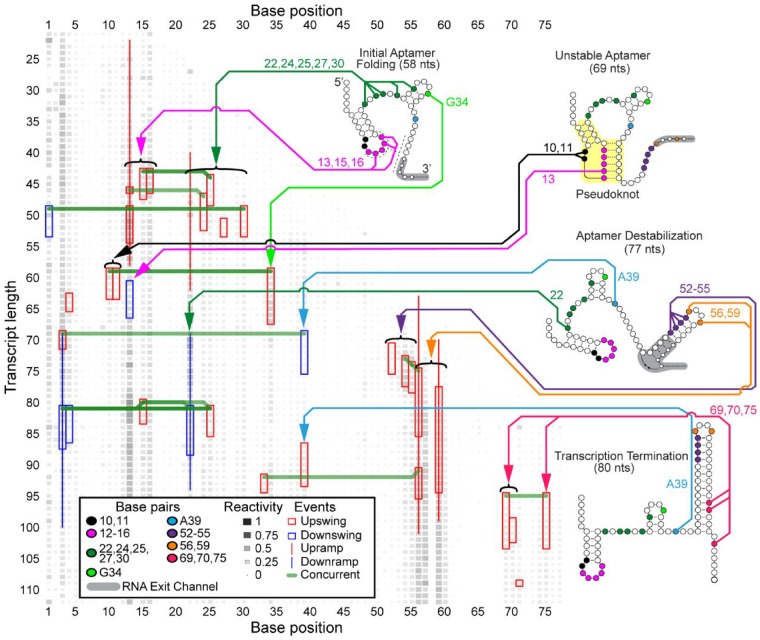
DUETT identifies changes in the *B.cereus* fluoride riboswitch with 0 mM fluoride cotranscriptional SHAPE-Seq reactivity matrices. When comparing to events identified with the fluoride added condition (Fig. 4), DUETT identifies multiple known and novel reactivity events, indicated by arrows to nucleotides participating in these events. The figure is annotated as in Figure 2. RNA structures and pseudoknot (highlighted in yellow) are redrawn from Figure 6 of Watters *et al.* (2016b)

**Fig. 4.**
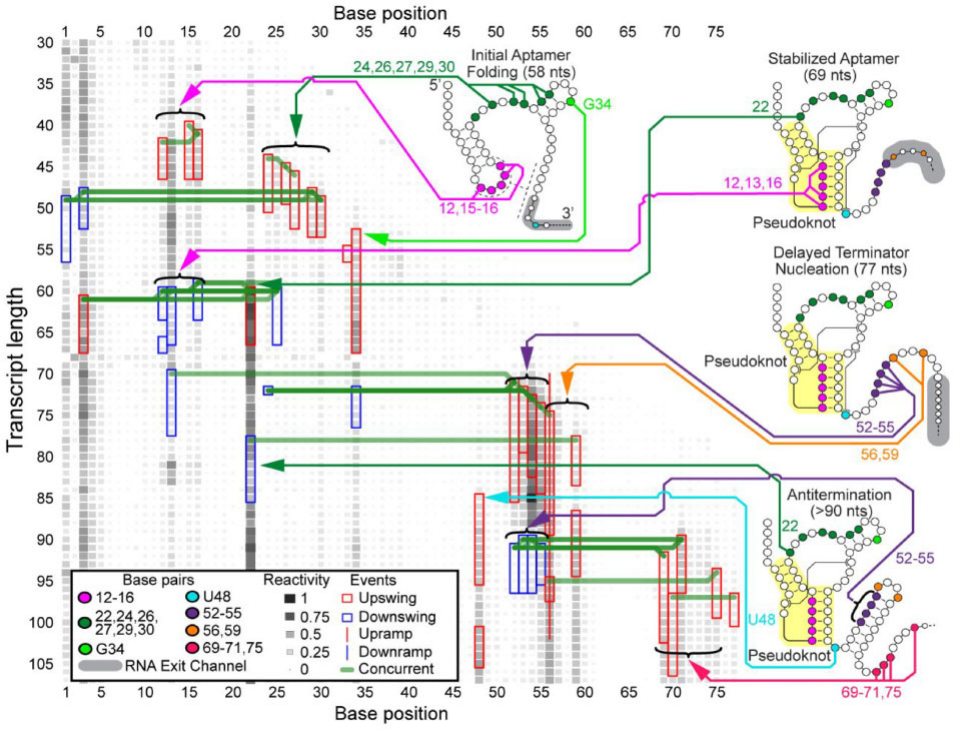
DUETT identifies changes in the *B.cereus* fluoride riboswitch with 10 mM fluoride cotranscriptional SHAPE-Seq reactivity matrices. These results are compared to Figure 3 to identify structural divergences between the fluoride conditions. The figure is annotated as in Figure 3

Before the structural divergence at the nascent RNA length of 69 nt, DUETT-detected events agree with the proposed model that RNA structures are similar in both fluoride conditions (Watters *et al.*, 2016b). Upswings in both conditions occur between 40 and 55 nt in bases 15–16, 24, 27 and 30 (Figs 3 and 4 and Supplementary Figs S7–S10), which confirm their unpaired state. Additionally, in the 10 mM fluoride condition, bases 12–13 and 16 have downswings around length 60 that suggest they form a critical pseudoknot, in which two helices are interleaved. Only base 13 has a downswing around length 60 in the 0 mM fluoride condition, which could indicate a less stable pseudoknot and thus a less stable aptamer. Base 30 has a consistent upswing in both conditions between lengths 49 and 53 nt, and the previously proposed model (Watters *et al.*, 2016b) suggests that bases 28–30 are paired within a hairpin stem spanning nucleotides 28–37 before length 58 nt. This base 30 upswing occurs when the hairpin stem theoretically forms (lengths 51–54) and may indicate delayed hairpin stability due to its short 3 bps length. The detected events before length 69 nt reflect similar RNA structures that form independent of the presence of fluoride (Watters *et al.*, 2016b).

#### 3.3.2 Identification of delayed terminator nucleation agrees with the previous riboswitch folding model

A key feature of the *B.cereus crcB* fluoride riboswitch folding pathway uncovered in previous work is a delay in the folding of the terminator RNA structure when fluoride is present (Watters *et al.*, 2016b). Correspondingly, DUETT identified events that agree with this delayed terminator nucleation. Exclusively in the 0 mM fluoride condition, bases 12–16 were previously proposed to unpair by length 77 nt, as well as bases 52–55 pairing with bases 60–63 at length 77 nt (Watters *et al.*, 2016b). In both fluoride conditions, bases 52, 54 and 55 exhibit upswings around lengths 70–75 nt. However, the 0 mM fluoride bases immediately decrease in reactivity (upon pairing) while the 10 mM fluoride bases retain high reactivity (Supplementary Files 2 and 3). This delayed terminator nucleation in the 10 mM fluoride-positive condition manifests as a series of downswings around length 90 nt exclusively in the fluoride-positive condition, which corresponds to forming the hairpin stem, but only after a delay of about 10 nts transcribed (Watters *et al.*, 2016b). In addition, bases 56 and 59 exhibit upswings/upramps in both conditions, corroborating their unpaired nature within the loop of the terminator hairpin.

DUETT identified expected events that occur after terminator formation, when transcription is expected to halt exclusively in the 0 mM fluoride condition (Ren *et al.*, 2012; Watters *et al.*, 2016b). As expected by their reactive nature in the 10 mM fluoride condition (Fig. 4), bases 69–71 and 75, that compete for binding with nucleotides within the pseudoknot, remain unpaired and contain upswings shortly after transcription. These upswings are expectedly missing in the 0 mM fluoride condition except for bases 69, 70 and 75 (Fig. 3), which exhibit unexpected upswing at length 95, 99 and 95 nt, respectively. These events may have occurred because the RNAP transcribed past the expected termination site in the bulk cotranscriptional SHAPE-Seq experiment.

It was previously shown that the double mutant G69A, A70U prevents formation of the terminator stem, meaning that their pairing with bases 46–45 within the pseudoknot is a requisite of termination (Baker *et al.*, 2012; Watters *et al.*, 2016b). A separate study, using a similar riboswitch sequence, found that a single long-range reverse Hoogsteen base pair in the region shared by the aptamer and terminator stem area is pivotal in functional switching between termination and antitermination (Zhao *et al.*, 2017). These findings, coupled with the base 69 and 70 upswing in the 0 mM fluoride condition, suggest that a subpopulation of fluoride riboswitch RNAs do not form the base pairs, leading to imperfect termination and therefore increased reactivity in this region. However, the mechanism is unclear and requires further study. This previously overlooked observation demonstrates DUETT’s ability to identify interesting events for follow-up analysis.

#### 3.3.3 Detected events in bases 10 and 48 confirm long-range interactions

DUETT corroborates two previously reported long-range interactions in the 10 mM fluoride condition: A10-U38 and A40-U48 (Watters *et al.*, 2016b). These interactions were hypothesized to increase stability of the aptamer and persist only when fluoride binds (Watters *et al.*, 2016b). In the 0 mM fluoride condition, we observe an upswing in both bases 10 and 11 at length 60 nt, which corresponds to the opening of the initial hairpin to enable pseudoknot formation (Fig. 3). Conversely these upswings are absent in the 10 mM fluoride condition because the A10-U38 interaction prohibits increased SHAPE reactivity when ligand is present (Fig. 4). The other long-range interaction, A40-U48, was proposed to unpair between the lengths 77 and 88 nt upon terminator hairpin nucleation (Watters *et al.*, 2016b), which we also observe with an upswing in U48 at length 85 nt in the 10 mM fluoride data (Fig. 4).

Additionally, A39 is situated between these two long-range interactions and exhibits an unexpected downswing and upswing in the 0 mM fluoride condition at 69 and 87 nt, respectively. After 77 nt, A39 exhibits structural divergence with an unexpected upswing at 87 nt. Previous NMR characterization of this system showed that A39 (A35 in their numbering) undergoes local structural dynamics when no fluoride is bound and is stabilized when fluoride is bound (Zhao *et al.*, 2017). These swing events may reflect those local structural changes. Conversely, the 10 mM fluoride condition lacks this upswing most likely due to the neighboring A10-U38 long-range interaction and the structural context of the fluoride-bound aptamer. Altogether, DUETT detects swing events in bases 10–11, A39 and U48 that are consistent with the proposed aptamer stabilization via long-range interactions.

#### 3.3.4 DUETT more precisely identifies A22 dynamics

DUETT identified SHAPE hyperreactivity in A22 via an upswing at length 60 nt in the fluoride-positive condition, which was associated with aptamer stabilization due to ligand binding (Watters *et al.*, 2016b). This upswing is followed by a sharp downswing at length 78 nt, while another upswing shortly afterwards was undetected. The second upswing went undetected due to the short duration of the previous downswing, which causes high and low-reactivity positions to lump together during the sliding window averaging. Afterwards, the reactivity plateaus at a high value comparable to length 69 nt. The fluoride-negative condition has similar dynamics but are less extreme and were detected as ramps (Supplementary Files 2 and 3) demonstrating that swing and ramp events differentiate small from large changes as intended. We conclude that base 22 has similar dynamics (except in magnitude) across both conditions until about length 90 nt where only the 10 mM fluoride condition exhibits the rebound upswing. The difference in magnitude of A22 reactivity between the conditions was previously concluded to be indicative of ligand-mediated aptamer stabilization and destabilization, respectively (Watters *et al.*, 2016b). The 0 mM fluoride downswing was thought to be due to aptamer destabilization caused by the invading terminator hairpin formation. The analogous 10 mM fluoride-positive downswing could be a result of the imperfect nature of the switch causing some complexes to terminate even with fluoride, or due to the experimental setup that stalls RNAP complexes near the termination site that can allow the terminator to form. This complex behavior of A22 cannot be visualized in reactivity matrix figures due to the visual upper limit (set as a reactivity value of 4) (Watters *et al.*, 2016b). While the upper limit simplifies data visualization, DUETT accounts for all magnitudes and is partially insulated from disadvantages in human visualizations. Altogether, DUETT identified several expected structural differences between the fluoride conditions, and we generate multiple hypotheses to explain unknown or unexpected events.

### 3.4 Threshold parameters confer a tradeoff between true positive and false positive/negative events

DUETT balances the detection of true positive events with detection of erroneous false positive/negative events. Determining this balance highlights user preferences; if identifying small magnitude events is prioritized, then thresholds can be relaxed to increase finding true positive events at the cost of also increasing the number of false positives.

Parametric sensitivity analysis explores the true positive-false positive tradeoff. We demonstrate that large events are retained despite drastic threshold parameter adjustments. We highlight two scenarios in the *E.coli* SRP RNA dataset using a stringent and a lenient set of *PIR* thresholds. The stringent *PIR* scenario (100% increase in each threshold) yielded fewer overall events (Supplementary Fig. S11, right) relative to the original baseline (center). False positive events, such as the event in base 44 that arose from a single anomalous replicate, are removed. Similarly, qualitatively small true positives are also removed: the downswing at length 88 nt in base 14 and the downswings around 100 nt in bases 28–29. We previously observed that base 14’s downswing is likely non-spurious and marks a new discovery. Similarly, the downswings in bases 28–29 are attributed to their pairing off before the final structure. These removals underscore the tradeoff that while higher thresholds lower false positives, they also turn true positives into false negatives. This is also true for perturbations to single parameter values such as *I* length (Supplementary Fig. S13). We chose a large increase but retained many of the originally detected events, suggesting that large events have a wide acceptable range of threshold values.

Conversely, the lenient scenario (Supplementary Fig. S11, left; 50% decrease in each threshold) leads to more true and false positives. The upswings in bases 26 and 33 around length 90 nt become detected. By inspection, these events seem non-spurious and occur concurrently with other similar events (Supplementary File 1) leading to the conclusion that these are previously undetected true positive events. Conversely, the lenient scenario creates potential false positive events. For example, the upswings in base 40 at 118 nt and all upswings in base 44 do not resemble upswings. The upswings in base 44 are especially misleading; one replicate has increased reactivity while the others remain flat (Supplementary Fig. S11). We conclude that the lenient scenario reveals spurious events. Similar results were seen when analyzing sensitivity of window length choice (Supplementary Fig. S12) and DWS cutoff (Supplementary Fig. S14).

We chose drastic perturbations to threshold parameter values to interrogate their effect on detection rates. Many originally detected events remained in the stringent scenario and relatively few spurious events arose in the lenient scenario, suggesting that our methodology yields concordant results across a wide acceptable range of thresholds. We provide additional sensitivity analysis on window length and linear ramp thresholds in Supplementary Materials.

### 3.5 Global sensitivity test demonstrates DUETT’s ability to resolve reactivity changes

A pair-wise sensitivity test of the automated threshold selection for swing events demonstrates DUETT’s ability to resolve reactivity changes. All possible pairs of the seven PIR thresholds were tested on the SRP RNA dataset for four variants of 100% increase and 50% decrease (↑↑, ↑↓, ↓↑, ↓↓) for a total of 84 combinations. Supplementary Figure S15 shows the upswing/downswing agreement between the automated DUETT run and the cohort of 84 sensitivity tests. From visual inspection, events originally detected by the automated method agree with the sensitivity tests; most automatically detected events are also detected in at least 50% of the sensitivity tests (Supplementary Fig. S16). In contrast, the events detected in the sensitivity tests but not the automated method cluster around the originals in terms of transcript length, suggesting that DUETT correctly identifies structurally relevant nucleotides but not necessarily at the exact transcript length depending on parameter settings. This difference in transcript length assignments of events likely stems from the window averaging that is needed to attenuate noise as well as the tendency for structural events to occur over multiple transcript lengths. Other disagreements occur on nucleotides or nucleotide regions with especially high-reactivity changes, suggesting that DUETT is appropriately sensitive to structurally relevant RNA regions and not to nucleotides constrained in a stable structural element. For example, nucleotides 57–59 are located in the open region of a hairpin and contain detected events exclusive to the sensitivity tests, but the paired adjacent nucleotides do not contain detected events at all (Supplementary Fig. S15).

### 3.6 Experimental data considerations

Within the cotranscriptional SHAPE-Seq datasets, many upramps (positive-slope ramps) begin near the 3' end of the nascent RNA shortly after the nucleotide’s transcription by RNAP (Figs 2–4; Supplementary Figs S3, S11, S12 and S14). This close association suggests SHAPE adduct formation occurs almost immediately after exiting RNAP. Due to experimental limitations (Strobel *et al.*, 2018), these short RNA fragments are difficult to detect, leading to reduced reactivity signal. However, as the RNA elongates, SHAPE adducts at these same positions become increasingly detectable, which could lead to the presence of gradually increasing reactivity upward ramps in these regions. As a result, we infer that upramps close to the nucleotide’s exit from RNAP are likely experimental artifacts due to their position near the 3' end of the RNA. Similarly, DUETT also identifies changes where *ρ* reactivities increase at unpaired positions as RNAs elongate due to the normalization used in their calculation (Supplementary Fig. S4). In some cases, DUETT is likely to miss true reactivity changes at short transcript lengths because there is insufficient data before the reactivity change, or when events in the same nucleotide occur closely in terms of transcript length.

## 4 Conclusion

DUETT emulates human visual inspection of cotranscriptional SHAPE-Seq data in an automated, efficient and systematic manner to reduce potential user bias in discovering novel events that are difficult to manually detect. Cotranscriptional SHAPE-Seq complements information about aptamer structure and aptamer-ligand interactions by adding dynamical information about RNA structural rearrangements during nascent RNA folding. Because DUETT focuses on event detection from reactivity changes, our method in its current state is meant to aid in hypothesis testing and data visualization by the user. However, DUETT can be considered as a first step toward computationally automated model generation of low resolution cotranscriptional folding pathways by identifying important regions of the RNA that undergo large transitions. We believe future tools can further integrate DUETT’s output with other RNA folding algorithms and experimental datasets to create computationally automated higher-resolution model generation of cotranscriptional RNA folding at the nucleotide level. When interpreting cotranscriptional SHAPE-Seq data, it is also important to keep in mind that halted nascent RNA structures are probed and fleeting structural changes are difficult to detect. As we demonstrate, DUETT detects many of the previously identified signatures of nascent structures within three model systems and identifies several new events absent from manual visual analysis. Experimentalists can now quickly establish transcription lengths and nucleotides of interest from reactivities to be further interpreted and developed into a structural model. Additionally, the automated analysis allows experimentalists to use their reactivity measure of choice. In this study, we chose *ρ* reactivities for the ability to compare reactivities between different length transcripts and across different experiments. We note that correlations within a *ρ* reactivity vector make interpretation of detected events harder, especially when concurrent up and down events occur, as it could be reflective of either structural changes or reactivity calculation. We hope this method is readily adopted when studying new RNA systems, or interrogating publicly available datasets in the RNA Mapping DataBase (Cordero *et al.*, 2012) where reactivity changes over a continuously changing variable such as increasing transcript length in cotranscriptional SHAPE-Seq data, ligand concentration or temperature is an important aspect of the RNA system studied. DUETT is a powerful method to rapidly identify structural events that evade manual identification in cotranscriptional SHAPE-Seq data.

## Funding 

This work was supported by New Innovator Award through the NIGMS of the National Institutes of Health [1DP2GM110838 to J.B.L.]; Searle Funds at the Chicago Community Trust (to J.B.L.); the Center of Cancer Nano-technology Excellence initiative of the NIH’s National Cancer Institute [U54 CA199091 to N.B.]; and Northwestern University’s Data Science Initiative Award (to N.B.). Support was also provided by the Northwestern University Graduate School Cluster in Biotechnology, Systems, and Synthetic Biology (to A.Y.X.); and Tri-Institutional Training Program in Computational Biology and Medicine [NIH training grant T32GM083937 to A.M.Y.].

*Conflict of Interest*: none declared.

## Supplementary Material

btz449_Supplementary_Data
