# Supplementary material for: DUETT quantitatively identifies known and novel events in nascent RNA structural dynamics from chemical probing data
Source: Bioinformatics. 2019 Aug 7;35(24):5103–12. doi: 10.1093/bioinformatics/btz449 (PMC6954663; doi:10.1093/bioinformatics/btz449)
Supplement: btz449_Supplementary_Data [file btz449_supplementary_data.zip › btz449-Suppl_data/Supplementary_File_2.pdf]

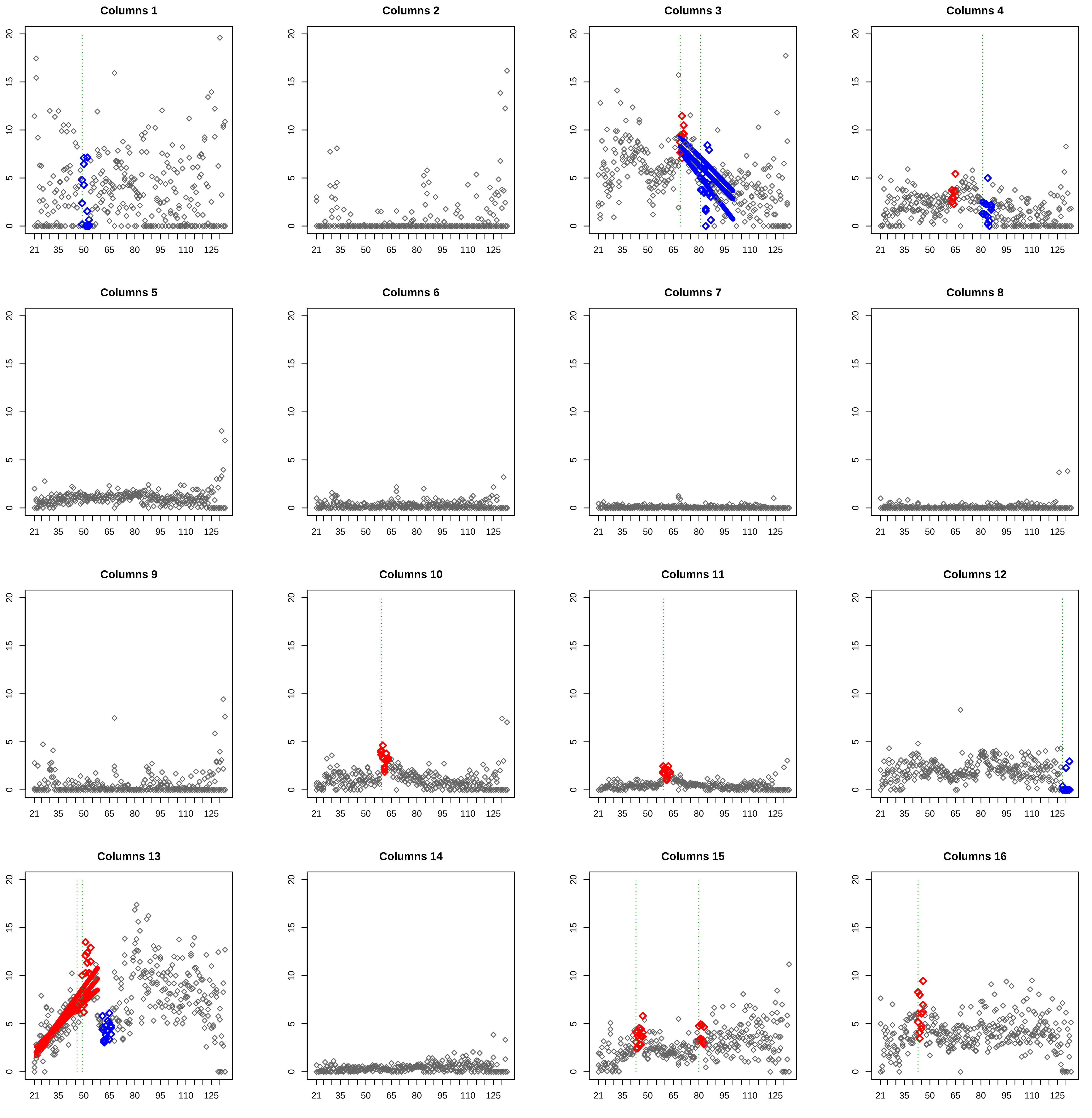
Columns 1
Columns 2
Columns 3
Columns 4
Columns 5
Columns 6
Columns 7
Columns 8
Columns 9
Columns 10
Columns 11
Columns 12
Columns 13
Columns 14
Columns 15
Columns 16
0 5 10 15 20
21 35 50 65 80 95 110 125

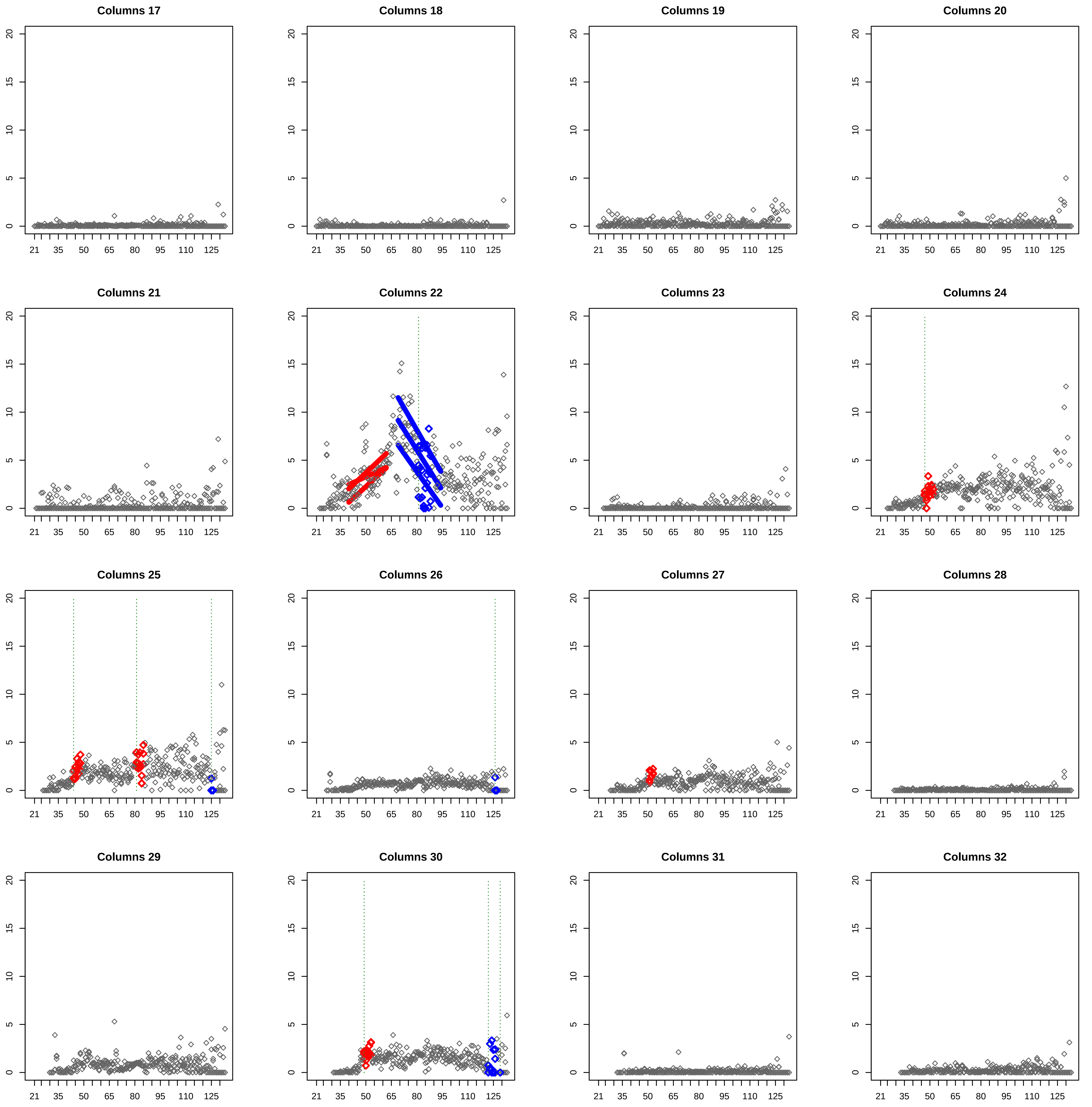
Columns 17
Columns 18
Columns 19
Columns 20
Columns 21
Columns 22
Columns 23
Columns 24
Columns 25
Columns 26
Columns 27
Columns 28
Columns 29
Columns 30
Columns 31
Columns 32
0 5 10 15 20
21 35 50 65 80 95 110 125

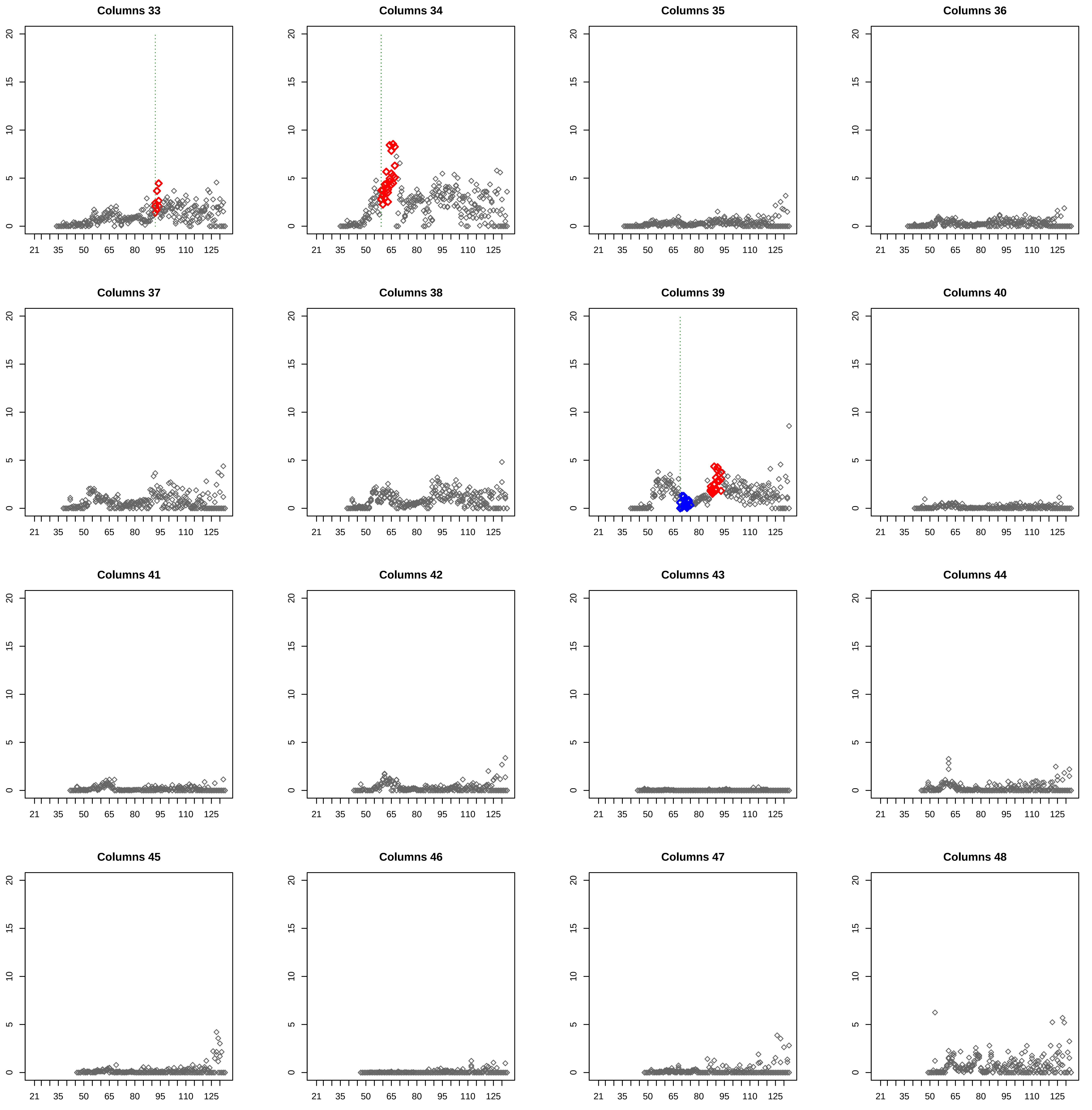
Columns 33
Columns 34
Columns 35
Columns 36
Columns 37
Columns 38
Columns 39
Columns 40
Columns 41
Columns 42
Columns 43
Columns 44
Columns 45
Columns 46
Columns 47
Columns 48
0 5 10 15 20
21 35 50 65 80 95 110 125

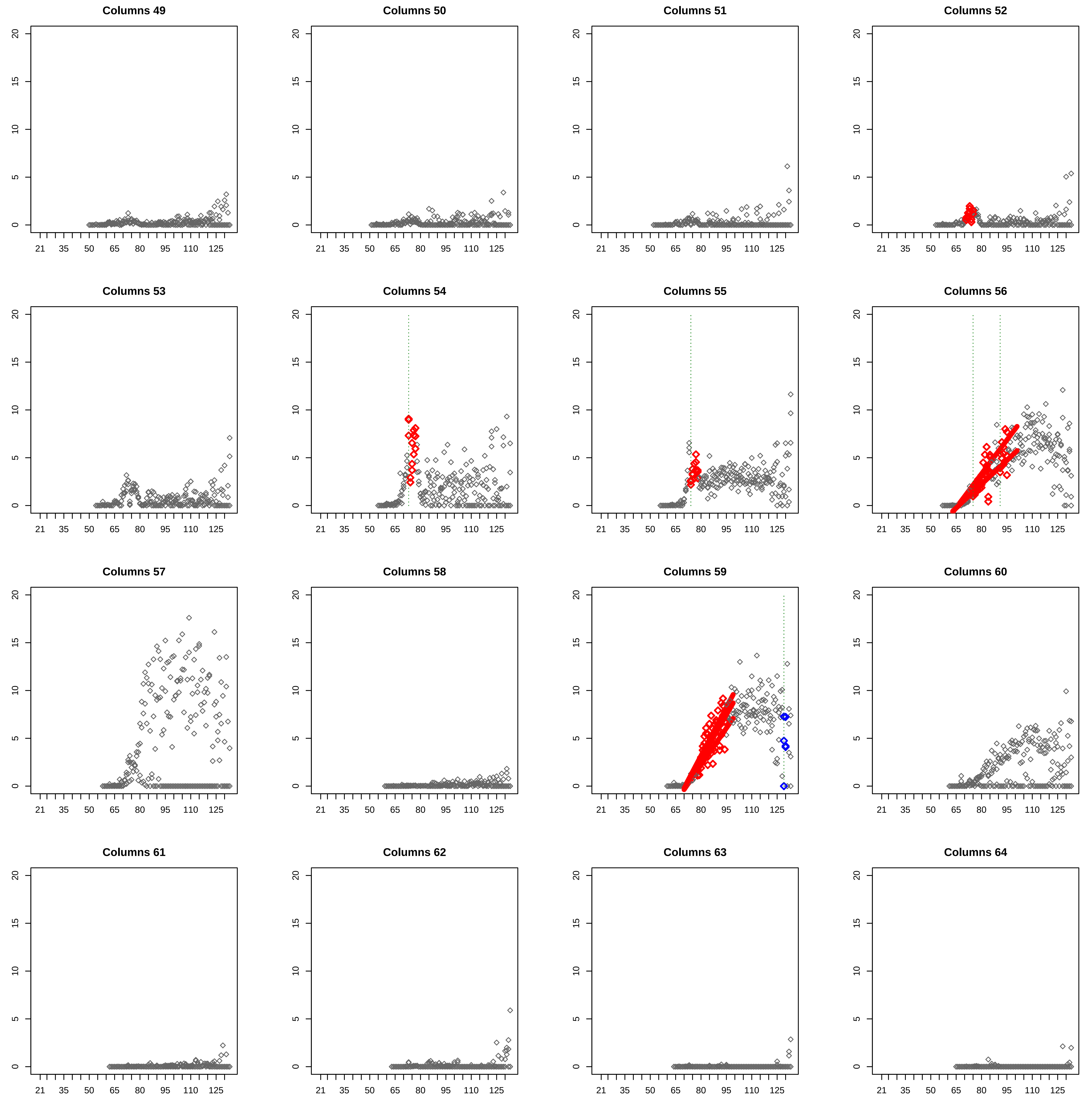

Columns 49
Columns 50
Columns 51
Columns 52
Columns 53
Columns 54
Columns 55
Columns 56
Columns 57
Columns 58
Columns 59
Columns 60
Columns 61
Columns 62
Columns 63
Columns 64
0 5 10 15 20
21 35 50 65 80 95 110 125

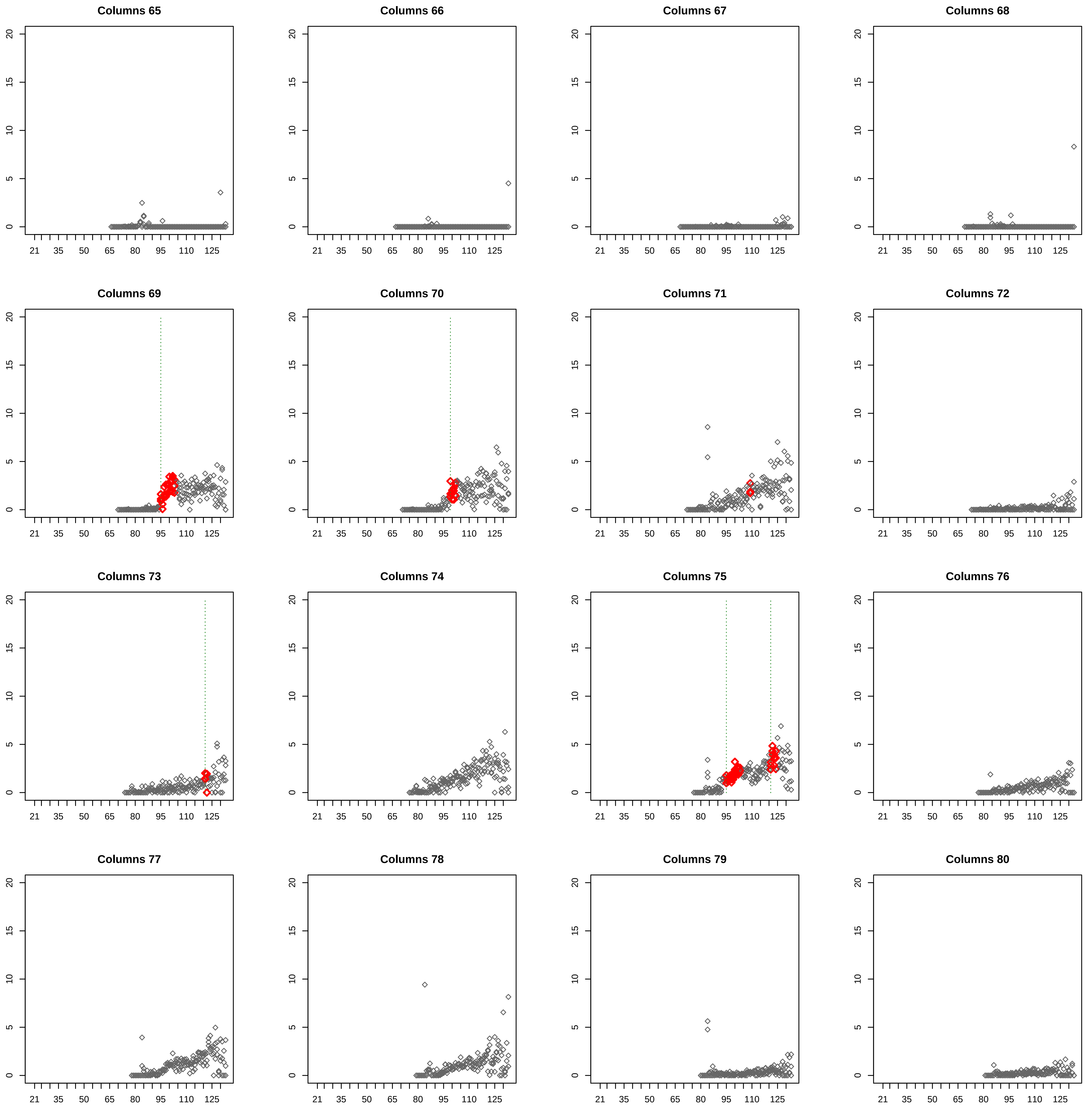
Columns 65
Columns 66
Columns 67
Columns 68
Columns 69
Columns 70
Columns 71
Columns 72
Columns 73
Columns 74
Columns 75
Columns 76
Columns 77
Columns 78
Columns 79
Columns 80
0 5 10 15 20
21 35 50 65 80 95 110 125

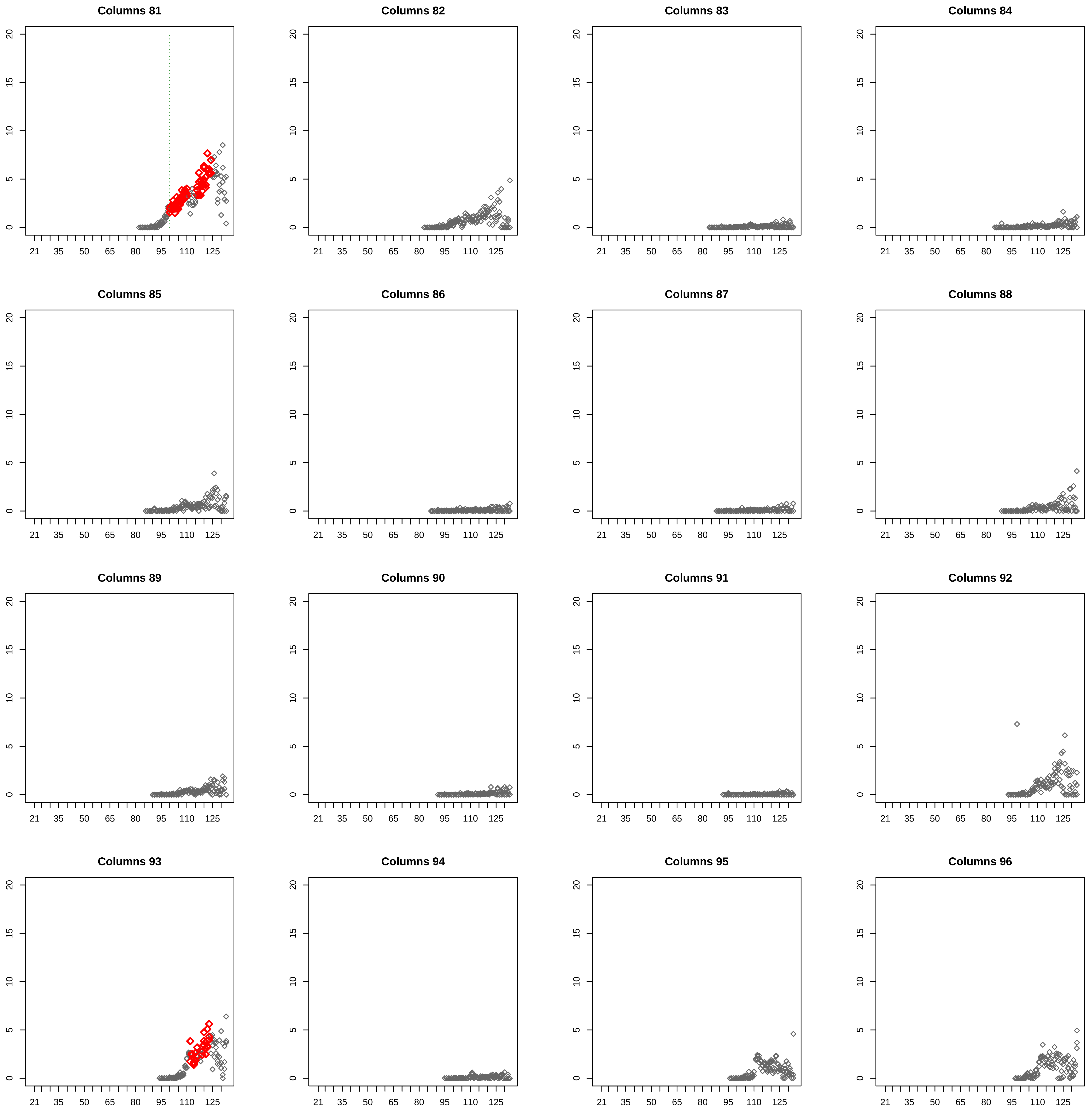
Columns 81
Columns 82
Columns 83
Columns 84
Columns 85
Columns 86
Columns 87
Columns 88
Columns 89
Columns 90
Columns 91
Columns 92
Columns 93
Columns 94
Columns 95
Columns 96
0 5 10 15 20
21 35 50 65 80 95 110 125

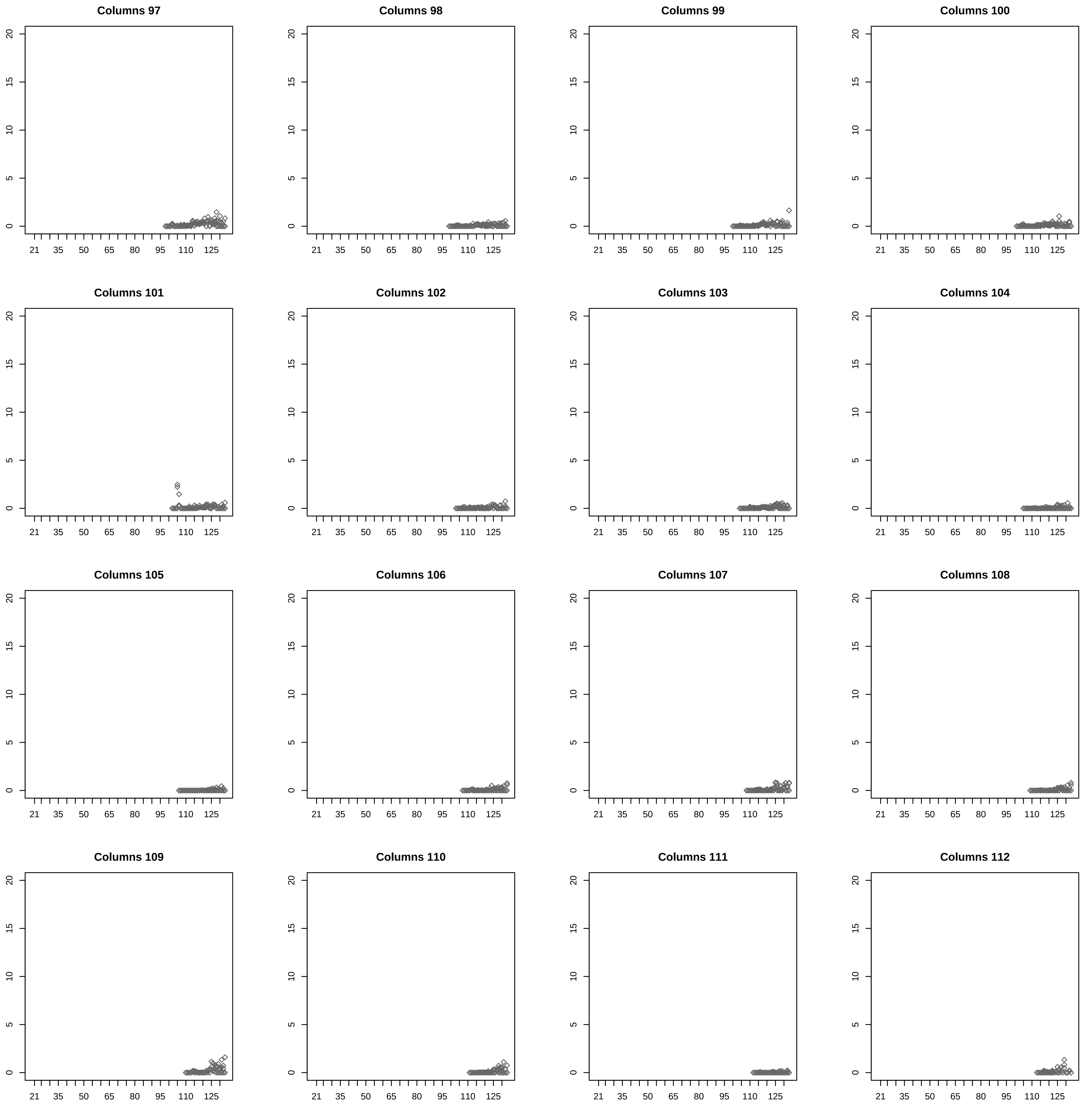
Columns 97
Columns 98
Columns 99
Columns 100
Columns 101
Columns 102
Columns 103
Columns 104
Columns 105
Columns 106
Columns 107
Columns 108
Columns 109
Columns 110
Columns 111
Columns 112
0 5 10 15 20
21 35 50 65 80 95 110 125

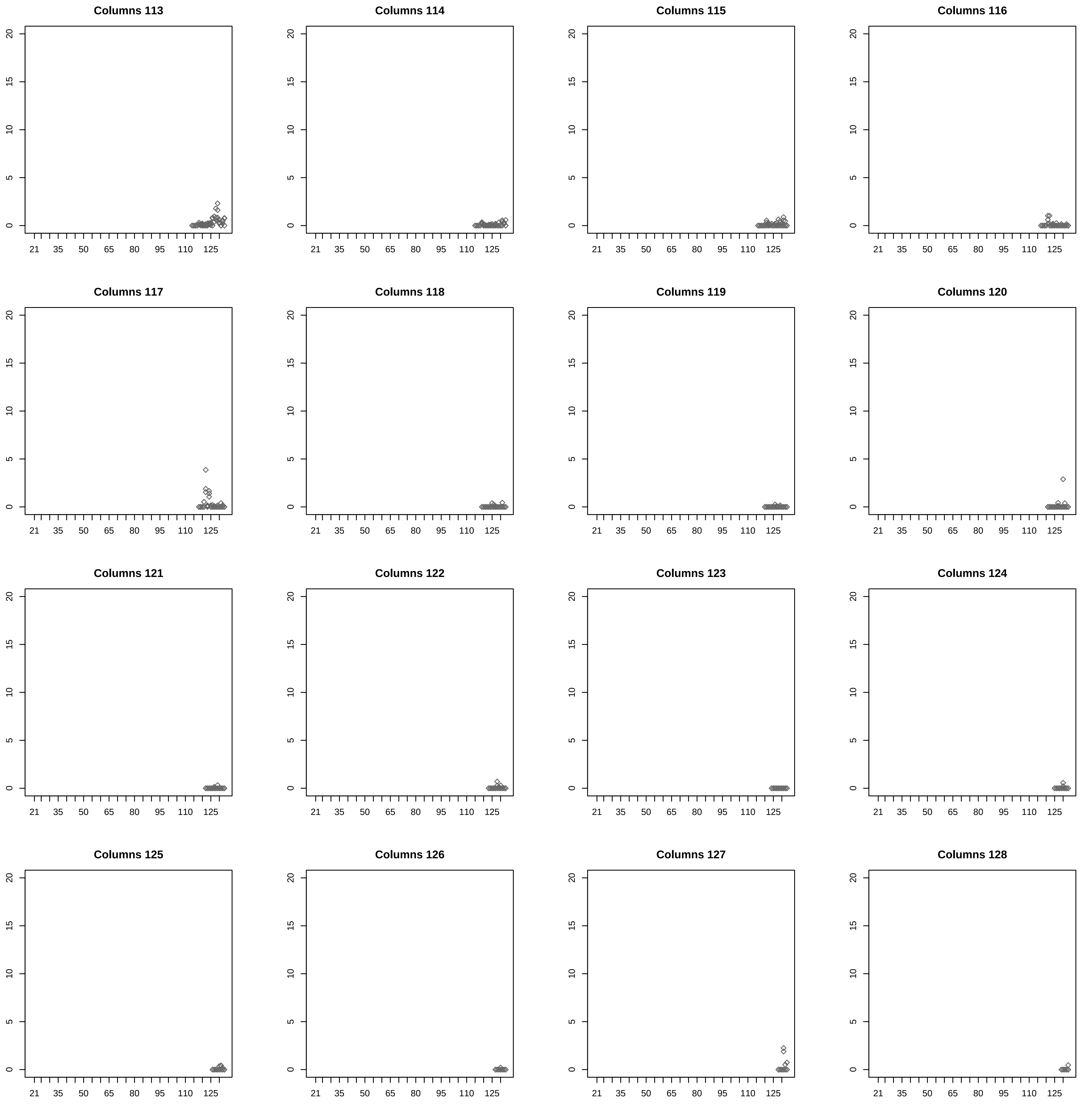
Columns 113
Columns 114
Columns 115
Columns 116
Columns 117
Columns 118
Columns 119
Columns 120
Columns 121
Columns 122
Columns 123
Columns 124
Columns 125
Columns 126
Columns 127
Columns 128

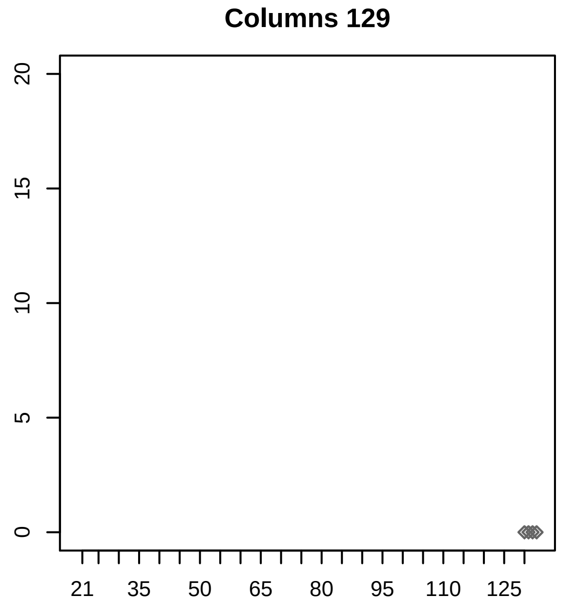
Columns 129
0
5
10
15
20
21
35
50
65
80
95
110
125

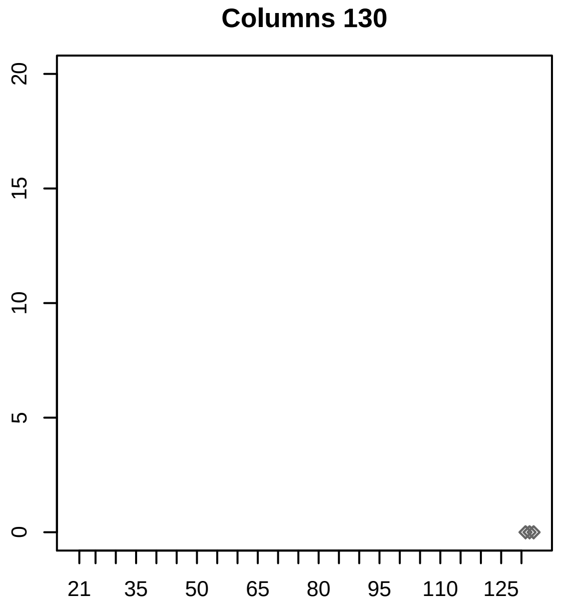
Columns 130
0
5
10
15
20
21
35
50
65
80
95
110
125

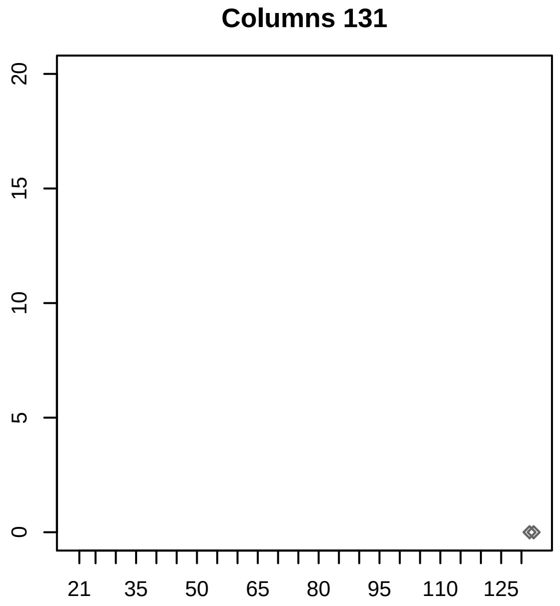
Columns 131
0
5
10
15
20
21
35
50
65
80
95
110
125

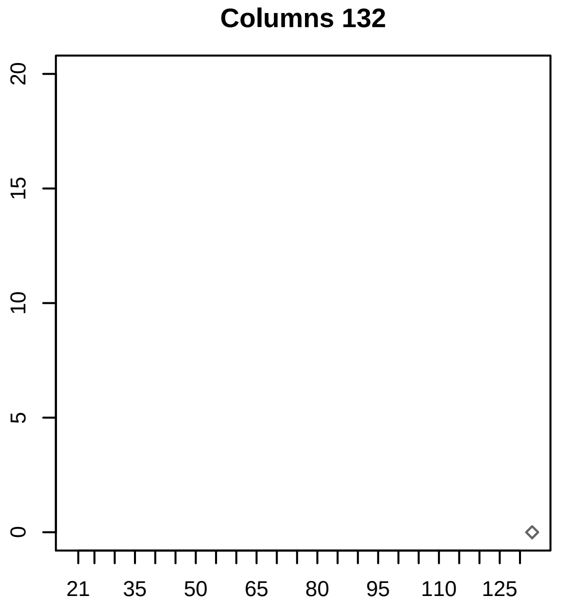
Columns 132
0
5
10
15
20
21
35
50
65
80
95
110
125
