# Supplementary material for: DUETT quantitatively identifies known and novel events in nascent RNA structural dynamics from chemical probing data
Source: Bioinformatics. 2019 Aug 7;35(24):5103–12. doi: 10.1093/bioinformatics/btz449 (PMC6954663; doi:10.1093/bioinformatics/btz449)
Supplement: btz449_Supplementary_Data [file btz449_supplementary_data.zip › btz449-Suppl_data/Supplementary_materials.docx.zip!/Supplementary_materials_v4.docx]

Supplementary Materials

**DUETT quantitatively identifies known and novel events in nascent RNA structural dynamics from chemical probing data**

Albert Y. Xue^1-3+^, Angela M Yu^2,4+^, Julius B. Lucks^1,2,5*^, and Neda Bagheri^1-3,5*^

^1^Department of Chemical & Biological Engineering, Northwestern University, Evanston, Illinois, 60208 United States

^2^Center for Synthetic Biology, Northwestern University, Evanston, Illinois, 60208 United States

^3^Northwestern Institute on Complex Systems, Evanston, Illinois, 60208, United States

^4^Tri-Institutional Training Program in Computational Biology and Medicine, Weill Cornell Medicine, New York, New York

^5^Chemistry of Life Processes Institute, Evanston, Illinois 60208 United States

+Equal contribution

*To whom correspondence should be addressed.

This supplementary file contains the methods for DUETT, results from the automated threshold selection process, results from a simulated dataset, pseudocolor and select trace reactivities for Figures 2-4, results from averaging replicates then conducting event detection, additional sensitivity analysis, a table of assumptions, and a table of user-defined threshold parameters used in the SHAPE-Seq event detector manuscript. This document also contains descriptions for Supplementary Files 1-3.

## DUETT methods

### Swing event detection is motivated by PI control

Feedback control is widely deployed throughout the process systems industries to mitigate fluctuations of key process variables about a desired system state (Minorsky, 1922). One common application is to maintain steady-state response by taking corrective action based on the measured deviation of controlled system variables from their nominal steady-state values. PI controllers scale the strength of their corrective action based on both the *proportion* ($P$) to, and *integral* ($I$) of, the measured deviation from steady state. The $P$ and $I$ terms provide a convenient framework for quantifying the magnitude and duration of the system’s deviation from steady state, which we use to detect significant SHAPE reactivity events.

Swing events are characterized by abrupt changes in SHAPE reactivity during transcript elongation. These events may be represented as *deviations* ($\Delta$) about a constant reactivity value:

$$\Delta_{i}=z_{i+1}-\bar{z}_{\left( i-n \right):i}$$

where $i$ indexes transcript length, $z$ is SHAPE reactivity, and $\bar{z}_{\left( i-n \right):i}$ is mean reactivity within a sliding window of length $n$ starting at position $i-n$ and ending at position $i$. DUETT quantifies the absolute magnitude and duration of these deviations at each transcript length by adopting both $P$ and $I$ terms:

$P_{i}=\Delta_{i}$,

$I_{i}=\frac{1}{n}\int_{i+1-n}^{i+1} \Delta_{i}$,

where $I$ is normalized by the window size. A third quantity captures the relative magnitude ($R$) of deviations:

$R_{i}=\frac{\Delta_{i}}{\bar{z}_{\left( i-n \right):i}}$.

DUETT detects an upswing when each of the $P$, $I$, and $R$ (PIR) values exceed set thresholds. Higher $P$ and $R$ thresholds require that reactivity changes are large and distinct enough from the local steady state (Supplementary Figure S1). Higher $I$ thresholds ensure that deviations reflect sustained reactivity changes rather than brief noise-driven fluctuations (Supplementary Figure S1). All threshold values are positive; a zero-value denotes constant reactivity.

Downswing events are detected similarly using the additive inverse of the *PIR* thresholds. The downswing $R$ threshold requires a slight modification to retain an equivalent magnitude to its upswing counterpart:

$R_{th,down}=-\left( \frac{R_{th,up}}{1+R_{th,up}} \right)$.

For example, an upswing event with 50% increase relative to the local steady-state reactivity ($R_{th,up}=0.5$) is of equivalent magnitude to a downswing event with 33% decrease ($R_{th,down}=0.33$).

We introduce two additional threshold parameters to further mitigate the impact of minor fluctuations.

**Figure S1. Three threshold parameters detect true positive events from true negative ones**. Swing and ramp events differ in terms of event length and have their own set of threshold parameters for detection. DUETT detects swing events using a strategy inspired by control theory where proportional ($P$) and relative ($R$) thresholds filter out noise at low and high magnitudes, respectively, and the integral ($I$) threshold filters out anomalous high values that persist for short durations. *In silico* illustrations and representative *PIR* values are shown for events in the region of interest (grey). DUETT detects ramp events using linear regression, where the ramp *p*-value ($p$), ramp slope ($\beta$) and Durbin-Watson statistic ($DWS$) thresholds filter out high noise, low slope ramps, and swing events that may be erroneously classified as ramps, respectively. We show *p*, $\beta$ and $DWS$ values for the fitted line (solid line). *p* is shown as -log_10_(*p*) so larger values pass the set threshold values (dotted line).

We remove swing events that are shorter than a specified *duration threshold* as we assume that structurally informative events generally persist for longer durations (Supplementary Table S1). Conversely, we merge swing events separated by a distance less than a *gap threshold* to reject noise-driven fluctuations that intersperse real swing events.

### Automated parameter selection for swing event detection

DUETT provides a method to automatically select *PIR* thresholds for any given dataset. Due to the subjective nature of event detection, the automated strategy relies on a heuristic similar to the elbow rule in cluster analysis (Ketchen and Shook, 1996). The heuristic identifies a threshold combination that balances lenient with stringent thresholds. The automated search starts with low PIR thresholds where both noise and real events are detected. DUETT scans over combinations of increasing *PIR* threshold values (within a user-defined range) and records the number of detected events. We expect a sharp decrease in the number of detected events as threshold values increase, followed by a leveling off, forming an elbow. DUETT identifies the vertex of the elbow—representing detection of true events—by finding the *PIR* threshold values that correspond to a detection output closest to the origin (Supplementary Figure S2). If needed, the automatically identified thresholds serve as a starting point for manual tuning.

### Ramp event detection using linear regression

Ramp events correspond to gradual changes in reactivity over broad stretches of sequential transcript lengths. The swing event detection method overlooks ramp events because it emphasizes rapid changes in reactivity over few transcript lengths. Instead, DUETT detects ramp events using a strategy based on linear regression. Given a user-specified *ramp length* corresponding to the minimum expected ramp length, lines of the form $y=\beta x$ are fit within windows sliding down each column of the SHAPE-Seq data matrix. Here, $x$ is a vector of sequential integers with length equal to the ramp length, $y$ is the measured reactivities within the corresponding window, and $\beta$ is a regression coefficient.

A ramp is detected when a fitted line passes three user-specified thresholds: a maximum *p-value* calculated from a one sample t-test for the regression coefficient, a minimum *regression coefficient* ($\beta$), and a minimum *Durbin-Watson Statistic* ($DWS$). Manual tuning of these thresholds is required to confidently detect ramp events. Fortunately, these parameters are readily interpretable (Supplementary Figure S1). The *p*-value threshold controls the robustness of event detection against measurement noise; low values improve specificity at the expense of sensitivity. The $\beta$ threshold constrains ramp steepness; high values exclude relatively flat ramp events with low average change in reactivity. Finally, the $DWS$ threshold tunes the selectivity of ramp versus swing event detection. Swing events yield strong positive autocorrelation among residuals about the regression line (Supplementary Figure S1, right panel of row 4), while residuals associated with true ramp events are uncorrelated. A $DWS$ threshold of unity precludes misclassification of swing events as ramps by filtering events whose sequential residuals are positively correlated (Nerlove and Wallis, 1966).

### Identifying concurrent events

Multiple events across different nucleotide positions (matrix columns) detected at similar transcript lengths likely reflect a common structural change. For example, a pair of upswings independently detected at two different nucleotide positions are involved in the same structural rearrangement if they occur at approximately the same transcript length. We label such instances as concurrent events by applying a *concurrency distance* proximity threshold.

### Computational development and graphical user interface

DUETT was programmed in the freely available statistical software environment R and RStudio. We provide the source code and a graphical user interface as an R Shiny app located at https://github.com/BagheriLab/DUETT. The app facilitates parameter tuning by continually updating figures as parameter values are varied by the user. The app also provides a suite of formatting tools for generating appropriate figures and tables.

**References**

Ketchen, D.J. and Shook, C.L. The application of cluster analysis in strategic management research: an analysis and critique. *Strategic Management Journal* 1996;17(6):441-458.

Minorsky, N. Directional stability of automatically steered bodies. *Journal of the American Society for Naval Engineers* 1922;34(2):280-309.

Nerlove, M. and Wallis, K.F. Use of the Durbin-Watson Statistic in Inappropriate Situations. *Econometrica* 1966;34(1):235-238.

**Figure S2. Automated *PIR* threshold selection identifies the balance between too lenient and too stringent parameter sets.** DUETT provides an automated method to select appropriate *PIR* thresholds for A) the SRP RNA, B) the fluoride riboswitch under 0mM NaF, and C) the fluoride riboswitch under 10 mM NaF datasets. After scanning over potential combinations of *PIR* threshold values, DUETT identifies the threshold set closest to the origin (red point with dotted line from origin). This point corresponds to the elbow criterion identifying the point when spurious detection is avoided while retaining true events. The horizontal axis is the sum of all three *PIR* thresholds and both axes are normalized. This calculation is done in 4-dimensions ($P$, $I$, $R$, and number of events) and the selected threshold values may not appear closest to the origin when plotted in 2D.

**Figure S3. Similar results are created when applying DUETT to the average of replicates.** Figure 2 shows results from identified events that are shared in each of the three replicate datasets on the SRP RNA. Here, all three replicates are averaged then event detection is conducted. The *PIR* thresholds are slightly more stringent than in Figure 2 because few events pass all thresholds and are conserved in all replicates. Each *PIR* threshold was increased by 0.1 from the values listed in Table S2.

**Figure S4. Application of DUETT to simulated cotranscriptional SHAPE-Seq reactivities.** A simulated cotranscriptional SHAPE-Seq dataset was created as described in Methods. The simulated folding pathway starts with an unstructured RNA, which then forms an initial hairpin at transcript length 36 (green highlight). This structure continues to grow in base pairs, first with the formation of an inner loop (purple highlight), and then subsequent extension of the base helix into the full hairpin (turquoise highlight). A second hairpin forms at transcript length 61, followed by a rearrangement into an extended helix with a different inner loop at length 67. Afterwards, base pairs are added to the base of the helix until the final structure is finished forming at length 84. Simulated reactivity data was generated as described in Methods, and structures represent minimum free energy structure predictions of the NUPACK-designed RNA sequence at each specified intermediate length. DUETT detected events corresponding to structural changes throughout this simulated folding pathway as indicated.

**Figure S5. Pseudocolor heatmaps of *E. coli* SRP RNA datasets.** Replicates are plotted separately: **(A)** replicate 1, **(B)** replicate 2, and **(C)** replicate 3.

**Figure S6. Select traces of reactivities found in the *E. coli* SRP RNA cotranscriptional SHAPE-Seq datasets.** Replicate data are plotted on each scatterplot for base 14, 27, 36, 42, 57, and 86. Each title of these plots are colored according to labels in Figure 2. Points colored in red and blue indicate upswings and downswings, respectively. Upramps are indicated as red lines. The presence of concurrent events in other bases are denoted by vertical dotted green lines at the event found in these selected bases.

**Figure S7. Pseudocolor heatmaps of 0mM fluoride *B. cereus* fluoride riboswitch datasets.** Replicates are plotted separately: **(A)** replicate 1, **(B)** replicate 2, and **(C)** replicate 3.

**Figure S8. Select traces of reactivities found in the 0 mM NaF *B. cereus* fluoride riboswitch cotranscriptional SHAPE-Seq datasets.** Replicate data are plotted on each scatterplot for base 10, 13, 22, 34, 39, 54, 56, and 69. Each title of these plots are colored according to labels in Figure 3. Points colored in red and blue indicate upswings and downswings, respectively. Upramps are indicated as red lines and downramps are indicated as blue lines. The presence of concurrent events in other bases are denoted by vertical dotted green lines at the event found in these selected bases.

**Figure S9. Pseudocolor heatmaps of 10 mM fluoride *B. cereus* fluoride riboswitch datasets.** Replicates are plotted separately: **(A)** replicate 1, **(B)** replicate 2, and **(C)** replicate 3.

**Figure S10. Select traces of reactivities found in the 10 mM NaF *B. cereus* fluoride riboswitch cotranscriptional SHAPE-Seq datasets.** Replicate data are plotted on each scatterplot for base 12, 22, 34, 48, 54, 56, and 69. Each title of these plots are colored according to labels in Figure 4. Points colored in red and blue indicate upswings and downswings, respectively. The presence of concurrent events in other bases are denoted by vertical dotted green lines at the event found in these selected bases.

**Figure S11. Sensitivity analysis of user-defined threshold values illustrates the tradeoff between true positive and false positive/negative detection.** A) The original *PIR* thresholds (center) are compared to a lenient scenario (left, 50% decrease in all threshold values) and a stringent scenario (right, 100% increase in all threshold values) on the SRP RNA dataset. In general, lenient thresholds increase sensitivity towards small magnitude events, but false positive events are detected with greater likelihood. Conversely, stringent thresholds have fewer false positive events, but the likelihood of detecting false negative events increases. Large magnitude events tend to be insensitive to large threshold deviations. B) Individual nucleotide positions are highlighted where reactivity profiles of all three replicate datasets are shown. Events are annotated as in Figure 2 except that reactivities and swing events are shown with diamonds.

## Additional sensitivity analysis

### Window length

We explore how window lengths affect event detection. Generally, longer window lengths lead to higher sensitivity (more true and false positives) because reactivities from shorter/earlier transcript lengths affect the average window used in *PIR* equations. With a 100% longer window (Supplementary Figure S11 right), this effect is shown with base 14, resulting in detected upswings. As a tradeoff, spurious events are included such as the downswing in base 37 (Supplementary Figure S11 right). On the other hand, a shorter window generally decreases sensitivity (Supplementary Figure S11 left). The upswings in base 40 after 75 nt are no longer steep enough to be included and the overall number of swing events has decreased.

A longer window does not globally increase event detection sensitivity as evident in false negatives for downswings in base 11 at length 87 nt (Supplementary Figure S12). This observation is due to two effects. First, the transcript lengths before 87 nt have an upward trend, and the longer window length averages over lower reactivity transcript lengths and causes the downswing to appear less significant. Second, the integral length for $I$ defaults to the same value as the window length, and longer integral lengths lower sensitivity. Instead, specifying a shorter window length (4) for the $I$ length generally increases sensitivity and recapitulates the downswings in bases 11 and 14 (Supplementary Figure S13).

This analysis demonstrates that longer windows and shorter windows generally increase and decrease sensitivity, respectively, but scenarios exist that go against this rule. Our detailed analysis serves as a cautionary tale that there are tradeoffs between identifying false and true events.

### Durbin-Watson Statistic

We provide sensitivity analysis for the Durbin-Watson statistic ($DWS$). We recommend a default setting between 1 and 1.25 as lower values traditionally correspond to a positive correlation, and we test a lenient (0.1) and a stringent threshold (1.5). As expected, the lenient and stringent thresholds have more and fewer detected ramps, respectively (Supplementary Figure S14). The lenient threshold allows linear ramps where the residuals are not uniformly distributed down the length of the ramp. For example, the downramp in base 14 has points clustered above and below the ramp, and we argue that this pattern does not conform to our expectations of a ramp and appears more like a downswing (it is detected as a downswing). In contrast, the upramp in base 41 appears genuine but is removed with the stringent $DWS$. Though minor, the flat region around length 50 nt creates a sequence of negative residuals that correspond to a positive autocorrelation, which fails the stringent $DWS$. However, we observe that the upramp in base 42 is preserved because it lacks a flat region within the upramp as large as in base 41. These three examples showcase how the $DWS$ threshold parameter removes ramps that resemble swing events

Overall, we apply large threshold changes and most qualitatively large events are still identified in all scenarios. This demonstrates that large and clean events can be insensitive to threshold changes, and the true positive-false positive tradeoff is applicable to qualitatively small or noisy events. Consequently, it is likely that DUETT identifies large RNA structural events with similar accuracy as a human, but the detector can also systematically identify small events whereas a human might not. Similarly, RNA structural events are not clearly defined and encompass a degree of subjectivity meaning that tradeoffs between identifying true positives and avoiding false positives/negatives must be considered. Altogether, these complexities justify the need for our quantitative and systematic approach.

**Figure S12. Sensitivity analysis of window length**. Window length effect is shown A) in general and B) with specific examples on the SRP RNA dataset. The window length determines the number of positions that are averaged together before *PIR* calculation. Longer lengths generally lead to higher sensitivity of true positives because the longer window retains earlier reactivities. In contrast, shorter lengths are less sensitive and generally lead to fewer events and false positives/negatives.

**Figure S13. Higher** $\boldsymbol{I}$ **length generally lowers sensitivity and longer window length does not always raise sensitivity**. By default, $I$ length is the same as the window length (left) but can be specified separately (right). Lower and higher values of $I$ length generally increase and lower sensitivity, respectively. Examples are drawn from the SRP RNA dataset.

**Figure S14. Sensitivity analysis of the Durbin-Watson statistic**. $DWS$ ranges from 0-4 where 2 represents our ideal scenario of no autocorrelation in the residuals. We present effects of lower and higher $DWS$ thresholds and compare the quality of fitted linear ramps on the SRP RNA dataset. Generally, lower $DWS$ thresholds are more lenient where lines are fitted on less line-like segments. To simplify visual analysis, swing events were removed.

**Figure S15. Results of global sensitivity tests on the SRP RNA cotranscriptional SHAPE-Seq dataset.** A total of 84 sensitivity tests were conducted on all $PIR$ parameters and are compared with the automated event detection method. Upswings (A) and downswings (B) detected across the global sensitivity tests are shown in greyscale where shading denotes the proportion of sensitivity tests with the detected swing event. Swing events found in the original automated method are shown in red/blue boxes. The high overlap of shaded regions with originally detected events suggest that the automated method robustly detects RNA reactivity changes.

**Figure S16. Agreement of global sensitivity tests with automated method.** Each originally detected swing event is compared to the global sensitivity test results. For each original event, the proportion of sensitivity tests that found the same event is shown as a bar height on the vertical axis for upswing (red) and downwing (blue) events. The events are sorted from highest agreement to least. A majority of the originally detected events agree with >50% of the sensitivity tests, demonstrating that the global sensitivity tests agree strongly with the original automated detection.

**Table S1. Explicit assumptions and design implications in DUETT**

| # | Assumption | Design implication |
| --- | --- | --- |
| 1 | Low magnitude measurements have noise that appear large in a proportional sense | $P$ thresholds filter out low magnitude noise |
| 2 | High magnitude measurements have high magnitude noise that appear large in an absolute sense | $R$ thresholds filter out high magnitude noise |
| 3 | Real swing events persist for more transcript lengths than spurious swing events | $I$ and event length thresholds filter out short events |
| 4 | Real swing events may become fragmented due to noise | Merge together short but adjacent swing events via event gap parameter |
| 5 | True linear ramps have a clean and low noise ramp | Ramp p-value threshold filters out noisy ramps |
| 6 | True linear ramps have a non-zero slope | $\beta$ threshold (slope) filters out ramps with a near-zero slope |
| 7 | Ramp residuals should be uniform down the length of the ramp; Ramps should be longer than swing events | Durbin-Watson Statistic threshold filters out non-uniform residuals; avoids ramps fitted on swing events |
| 8 | Events on different nucleotides that are close in terms of transcript length are likely to be related | Concurrent events are identified within a specified difference in terms of transcript length |

**Table S2. Automated *PIR* and user-defined linear ramp threshold parameters for the SRP and fluoride riboswitch examples**

| SRP RNA settings | |  | F riboswitch settings (0 mM NaF left, 10 mM NaF right) | | | |
| --- | --- | --- | --- | --- | --- | --- |
| *PIR* | |  | *PIR* | | | |
| Window length | 9 |  | Window length | | 9 | 9 |
| $P$ | 0.3 |  | $P$ | | 0.35 | 0.3 |
| $I$ | 0.025 |  | $I$ | | 0.05 | 0.05 |
| $R$ | 0.1 |  | $R$ | | 0.025 | 0.025 |
| $I$ length | default |  | $I$ length | | default | default |
| Noise length | 4 |  | Noise length | | 4 | 4 |
| Event gap | 1 |  | Event gap | | 1 | 1 |
|  |  |  |  |  | |  |
| Ramp | |  | Ramp | | | |
| Ramp length | 30 |  | Ramp length | | 30 | 30 |
| *p*-value | 1.00E-04 |  | *p*-value | | 1.00E-04 | 1.00E-0.4 |
| $\beta$ | 0.15 |  | $\beta$ | | 0.15 | 0.15 |
| $DWS$ | 1.25 |  | $DWS$ | | 1.25 | 1.25 |
|  |  |  |  | |  |  |
| Concurrent distance | 1 |  | Concurrent distance | | 1 | 1 |

## Supplementary File descriptions

Supplementary Files 1-3 contain the profiles for each nucleotide in *E. coli* SRP RNA and the *B. cereus* crcB fluoride riboswitch datasets without fluoride and with 10mM fluoride, respectively. Upswings, downswings, upramps, and downramps are shown with red diamonds, blue diamonds, red lines, and blue lines, respectively. Concurrent events are denoted with a dotted green line.
